# Integrin-Targeting Peptides for the Design of Functional Cell-Responsive Biomaterials

**DOI:** 10.3390/biomedicines8090307

**Published:** 2020-08-25

**Authors:** Junwei Zhao, Federica Santino, Daria Giacomini, Luca Gentilucci

**Affiliations:** Department of Chemistry “G. Ciamician”, University of Bologna, via Selmi 2, 40126 Bologna, Italy; junwei.zhao2@unibo.it (J.Z.); federica.santino2@unibo.it (F.S.); daria.giacomini@unibo.it (D.G.)

**Keywords:** integrin ligands, tumor-targeting nanoparticles, peptide conjugates, RGD peptides, drug delivery, smart nanomaterials, cell adhesion, tissue engineering, regenerative medicine

## Abstract

Integrins are a family of cell surface receptors crucial to fundamental cellular functions such as adhesion, signaling, and viability, deeply involved in a variety of diseases, including the initiation and progression of cancer, of coronary, inflammatory, or autoimmune diseases. The natural ligands of integrins are glycoproteins expressed on the cell surface or proteins of the extracellular matrix. For this reason, short peptides or peptidomimetic sequences that reproduce the integrin-binding motives have attracted much attention as potential drugs. When challenged in clinical trials, these peptides/peptidomimetics let to contrasting and disappointing results. In the search for alternative utilizations, the integrin peptide ligands have been conjugated onto nanoparticles, materials, or drugs and drug carrier systems, for specific recognition or delivery of drugs to cells overexpressing the targeted integrins. Recent research in peptidic integrin ligands is exploring new opportunities, in particular for the design of nanostructured, micro-fabricated, cell-responsive, stimuli-responsive, smart materials.

## 1. Introduction

In the last 20 years, many studies focused on the implication of integrins in important cell activities, in the signaling from and to the cell, and in a variety of diseases. After the identification of the peptidic recognition sequences to different kinds of integrins, much effort has been dedicated to identifying peptidomimetic ligands with antagonistic properties, especially for cancer therapy, circulatory, or inflammatory diseases. These issues have been widely discussed earlier [1,2,3,4,5], and will be not proposed again herein. Despite good initial results in vitro and in animal models, clinical trials have not met the expectations. Nevertheless, peptidic integrin ligands still maintain a noteworthy appeal. This review aims at summarizing the more innovative applications of peptide-material conjugates: from nanoparticles (NPs) to new drug delivery systems, to functionalized surfaces, self-assembled monolayers (SAMs) and finally to smart and responsive materials. These conjugates are expected to allow a range of applications in theranostics, disease monitoring, regenerative medicines, and tissue engineering.

## 2. Integrins Structure and Functions

Integrins are cell adhesion receptors of soluble and insoluble glycoproteins of the extracellular matrix (ECM). These glycoproteins include collagens, fibronectins (FN), vitronectin (VT), laminins, fibrinogen (Fib), as well as cell surface receptors, e.g., the vascular cell adhesion molecule-1 (VCAM-1) and the intercellular adhesion molecule (ICAM). Furthermore, integrins are involved in the assembly of the actin cytoskeleton and signal transduction pathways of biological and cellular functions: cell adhesion, migration, proliferation, cell differentiation, and apoptosis [6].

The integrins are type I heterodimeric transmembrane receptors composed of an α and a β subunits connected by non-covalent bonds. In mammals, 18 α and 8 β subunits have been discovered, and to date are known to combine into 24 distinct heterodimers. In both α and β subunits, a large N-terminal extracellular domain, a single transmembrane domain, and a short C-terminal intracellular cytoplasmic tail, can be identified. The only exception to this rule is the β4 subunit, characterized by a long cytoplasmic portion (>1000 residues) [7].

**Figure 1:**
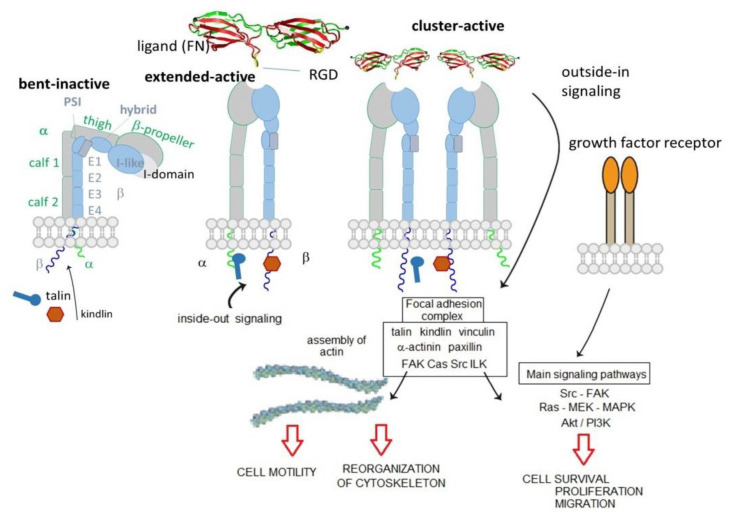
Bent-inactive, and open-active integrin states, and focal adhesions formation that consents outside-in downstream signaling.

The extracellular domain is composed of different highly organized subdomains, well-studied by high-resolution x-ray crystallography. The α subunit is formed by a β-propeller head domain, a thigh domain, and two calf domains. Some mammalian α subunits contain an additional portion called αA or I-domain. This last domain is very important because it contains the “metal-ion-dependent adhesive site” (MIDAS), which is the main domain responsible for ligand binding. When the I-domain is not present in the α subunit, the MIDAS is located inside an I-like domain of the β subunit. The I-like domain, together with a PSI (plexin/semaphorin/integrin) domain, a hybrid domain, four EGF repeats, and a B tail, constitute the β subunit [8] (Figure 1). 

The extracellular domain is responsible for ligand binding. Integrins can regulate their modules by shifting from a closed-resting structure to an open one that allows the binding and promotes the activation. The idle state consists of a bent conformation that straightens when the domain receives an impulse from within the cell (inside-out signal). The active conformation exposes the binding site and allows the ligand to connect and to activate the transmission of the signal inside the cell (outside-in signaling). Intracellularly, a large, dynamic multiprotein complex is formed, involving over 150 intracellular proteins, the so-called tight focal adhesions (FA), important for actin cytoskeletal assembly. Actin filaments are reorganized into larger stress fibers leading to further integrin clustering and enhanced matrix binding, allowing integrins to regulate cytoskeleton organization and cell motility. FAs are also responsible for the activation of further downstream signals to control various cellular functions like cell growth, apoptosis, cell proliferation, cell shape, and angiogenesis [8]. In 2017, Springer et al. demonstrate that ultrasensitive integrin activation depends on adaptor binding and cytoskeletal force, which was quantitatively evaluated by measuring the free energy of different conformational states of integrin [9].

## 3. Integrin-Correlated Diseases

As mentioned above, the different integrins are involved in a variety of diseases, including tumor metastasis, tumor invasion, angiogenesis, inflammation, thrombosis, osteoporosis, ocular diseases, restenosis, and autoimmune diseases, i.e., type-1 diabetes (T1D), multiple sclerosis (MS), rheumatoid arthritis (RA), and Crohn’s disease [10,11].

The integrin αIIbβ3 plays a key role in several diseases related to the cardiovascular system, like thrombosis, hemostasis, angina pectoris, and stroke. In particular, this heterodimer is responsible for platelet aggregation and adherence with soluble fibrinogen (Fib). As Fib molecules are bivalent, they can form an integrin-Fib-integrin bridge between adjacent platelets [12].

Another pathology correlated to integrin αIIbβ3 is restenosis [13,14], i.e., the lumen diameter reduction or re-occlusion after the cardiovascular intervention, angioplasty, or stenting, due to migration of vascular smooth muscle cells (SMCs) towards the site of injury. This migration is critically dependent on the interaction between the integrin receptors highly expressed on the surface of SMCs with extracellular matrix component osteopontin which is upregulated during restenosis.

The integrins of the α4 family are fundamental to the inflammatory processes, and in particular, α4β1 integrin is crucial for the recruitment of leukocytes [15]. In the proximity of inflammation, the local release of cytokines and chemokines stimulates adjacent endothelial cells to overexpress VCAM-1 on their vascular surfaces. The consequent production of chemoattractants and the activation of lymphocytes promote a conformational change that triggers integrin clustering along the contact surface. This movement rapidly strengthens integrin-ligand interaction and stops leukocytes rolling in the blood vessel. Once arrested, the immune cells can penetrate the underlying tissue (extravasation/diapedesis).

This process is also at the basis of many autoimmune disorders, including allergy, asthma, psoriasis, and Crohn’s disease. These disorders are characterized by an inappropriate inflammatory response against self, which ultimately results in damage to the affected tissue. Autoimmune diseases (AD) such as rheumatoid arthritis, autoimmune encephalomyelitis, and multiple sclerosis (MS), are characterized by α4β1 integrin-mediated invasion of “autoreactive” lymphocytes, while Celiac Disease is characterized by migration of T cells to the gut, guided by α4β7 integrins [16].

Osteoporosis is a degenerative disease characterized by low bone mass and bone degradation with resultant fragility and risk of fracture. Such a condition is caused by an increase in osteoclast differentiation and activity mediated by ανβ3 and ανβ5 integrins [17].

The progression of ocular diseases such as retinopathy of prematurity (ROP), age-related macular degeneration (AMD), and proliferative diabetic neuropathy (PDR), depends on retinal neovascularization. Interaction of ανβ3 and ανβ5 integrins with components of the modified ECM, such as vitronectin, leads to the successful neovascularization of the retinal surface, which eventually extends towards the vitreous region of the eye [18,19].

Furthermore, the engagement of integrins in cell activity, make ανβ3 and other heterodimers like ανβ5, α5β1, α6β4, α4β1, and ανβ6, responsible for development and progression of a large variety of cancers and in tumor angiogenesis [20]. Angiogenesis, i.e., the formation of new blood vessels, is a requirement for tumor growth and tumor metastasis. Moreover, the density of the microvasculature at the tumor site has been found to correlate well with the risk of distant metastasis.

## 4. Peptide and Peptidomimetic Integrin Ligands

Each integrin can bind a variety of ligands; on the other hand, one ligand, either a protein of the ECM or a cell surface adhesion protein, binds to multiple integrin receptors [6]. Recent structure-function analyses of both integrins and their ligands have revealed a similar mode of molecular interaction that explains this promiscuity. In 1984, Pierschbacher and Ruoslahti described the Arg–Gly–Asp (RGD) peptide motif as a minimal integrin recognition sequence within FN [21,22]. Later, this tripeptide was found to bind to several other integrins: ανβ1, ανβ3, ανβ5, ανβ6, ανβ8, α5β1, α8β1, and αIIbβ3. Other integrins interact with their ligands by recognizing different ligand motif. For instance, α4 integrins (α4β1 and α4β7) recognize Leu–Asp–Val–Pro (LDVP) motif in FN, Leu–Asp–Thr–Ser (LDTS) sequence in the mucosal addressing cell adhesion molecule-1 (MAdCAM-1), and Ile–Asp–Ser (IDS) in VCAM-1.

**Figure 2:**
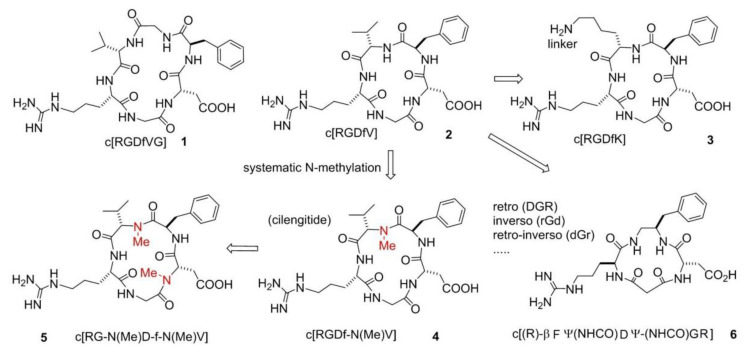
Examples of cyclopeptide integrin ligands: c[RGDfVG], and c[RGDfV], from which derive c[RGDfK], c[RGDf-N(Me)V], and c[(R)-βPheΨ(NHCO)AspΨ-(NHCO)Gly-Arg].

A systematic study conducted by Kessler and co-workers [23], the so-called spatial screening, allowed to investigate the 3D disposition of the pharmacophoric groups of the RGD sequence. In this context, two cyclic molecules emerged: c[RGDfVG] (**1**), an inhibitor of Fib from binding to the integrin αIIbβ3 (IC_50_ αIIbβ3/FB 5.0 × 10^−8^ M) and c[RGDfV] (**2**), which was shown to prevent the binding of VN to integrin ανβ3 (IC_50_ ανβ3/VN 4.9 × 10^−8^ M). Structure-Activity Relationships (SAR) studies revealed that Val was the less important residue and, because of that, it was replaced or modified. This lead to two important molecules, c[RGDfK] (**3**) and c[RGDf-N(Me)V] (**4**). The presence of a Lys allowed linking c[RGDfK] to other molecules or NPs or fluorescent probes, to create drug delivery or diagnostic systems (see next sections). c[RGDf-N(Me)V], or cilengitide, was renowned for its outstanding affinity (but not selectivity) for ανβ3 integrins (IC_50_ 6.5 × 10^−10^ M) (Figure 2). Further N-methylation yielded c[RG-N(Me)D-f-N(Me)V] (**5**), which showed a slightly decreased affinity for ανβ3 integrins (IC_50_ 5.9 × 10^−9^ M) but much higher selectivity over ανβ5 and α5β1 integrins. Starting from these cyclic structures, the retro (DGR), inverso (rGd), retro-inverso (dGr), and other peptidomimetic strategies [24,25,26] were extensively adopted (Figure 2). 

One meaningful example of peptidomimetic is the cyclotetrapeptide (CTP) c[(R)-βPheΨ(NHCO)AspΨ-(NHCO)Gly-Arg] (**6**), based on a partially modified retro-inverso (PMRI) variant of a CTP containing a β-amino acid, forming a 13-membered ring (Figure 2). This PMRI-CTP displayed a good activity in inhibiting the ανβ3 integrin-mediated cell adhesion of FN or VN (IC_50_ ανβ3/FN on SK-MEL-24 cells 1.8 × 10^−7^ M), as well as the adhesion of FN to the α5β1 integrin (IC_50_ α5β1/FN on K562 cells 2.4 × 10^−8^ M). This antagonist significantly inhibited bFGF-induced human endothelial cell tube formation at submicromolar concentrations [27]. Conformational analysis and Molecular Docking calculations suggested that integrin specificity can be rationalized based on the display of the aromatic side-chain adjacent to Asp.

In the last 20 years, the RGD and other integrin-targeting peptide sequences became the models from which a huge number of peptidomimetics and non-peptides were designed [28,29]. This wide topic has been adequately reviewed elsewhere [2,3,4,5,30]; hence, it will be not discussed further here. As for cilengitide, for its potent antagonism for pro-angiogenic integrins ανβ3, ανβ5 and α5β1, it became one of the most investigated anticancer peptides in vitro and in vivo. Preclinical in vitro studies confirmed that cilengitide was a potent inhibitor of angiogenesis, and promoted apoptosis in cancer cells obstructing the interaction between integrins and their ECM ligands [31,32]. Furthermore, numerous in vivo experiments confirmed the previous results supporting the ability of cilengitide to block tumor growth in a dose-dependent fashion [33]. After these promising results, phase I and II trials in solid tumors and glioblastoma started, with cilengitide alone or in combination with radiotherapy and/or chemotherapy with temozolomide [34,35,36,37]. As no dose-limiting toxicities (DLTs) were observed, the CENTRIC EIRTC phase II trial was started, but the latter lead to disappointing results. Cilengitide failed to demonstrate survival advantage and that it had a short plasma half-life. Subsequently, Massabeau, Khalifa, and co-workers [38] proposed continuous exposure for optimal efficacy. This idea was pursued in a phase I clinical study that combined cilengitide and chemotherapy for stage III NSCLC (non-small cell lung cancer) patients. The results evidenced the safety profile of the administration of cilengitide as continuous infusion and suggested possible future investigations. 

**Figure 3:**
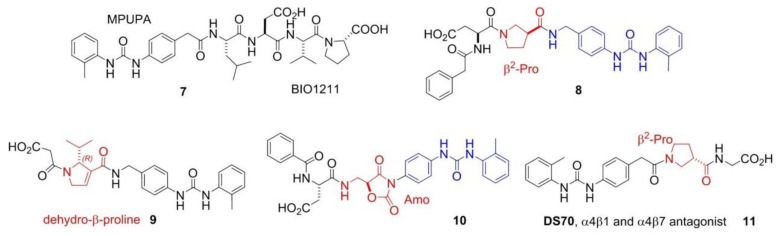
Examples of antagonist integrin ligands.

Despite of the (so far) failure for cancer treatments, integrin ligands remain privileged tools for other diseases. In particular, ligands that interfere with α4 integrin functions are currently investigated for inflammatory or autoimmune diseases correlated to leukocyte recruitment [1,39]. BIO1211 (**7**), MPUPA-Leu-Asp-Val-Pro-OH (MPUPA, o-methylphenylureaphenylacetic acid), (Figure 3) is a potent inhibitor of α4β1 integrin [40], which unfortunately is readily metabolized in heparinized blood, plasma and rat liver, lung and intestinal homogenates. Starting from the structure of BIO1211, Dattoli et al. designed an α4β1 integrin inhibitor (**8**) containing the pyrrolidine-3-carboxylate (β^2^-Pro) scaffold (Figure 3). The hybrid structure conferred to this compound good enzymatic stability, being degraded of only about 10% in mouse serum after 120 min. The peptidomimetic efficiently inhibited the adhesion of α4β1 integrin-expressing cell to VCAM-1 [41]. The retro-inverso approach was exploited to design BIO1211 peptidomimetics with a dehydro-β-proline core (**9**). The products showed to be effective and selective α4β1 integrin antagonists and displayed IC_50_ values in the nanomolar range in cell adhesion inhibition assays and VCAM-1-induced phosphorylation of extracellular-signal-regulated kinases. Significant activity was observed also toward the homologous integrin α4β7, but not toward the β1, β2, and β3 families [42]. Subsequently, a mimetic of BIO1211 containing a β^2^/α-hybrid Freidinger lactam analog (**10**) was proposed (Figure 3) [43], i.e., the aminomethyloxazolidine-2,4-dione scaffold (Amo) [44]. Interestingly, this mimetic displayed significant ability to inhibit the adhesion of α4β1 integrin expressing cells (IC_50_ α4β1/VCAM on Jurkat cells 1.9 × 10^−8^ M), and remarkable stability in mouse serum.

Finally, Dattoli et al. [45] reported the α/β hybrid-peptide DS70 (**11**) (Figure 3). The affinity for α4β1 integrin was determined in vitro in the scintillation proximity assay (IC_50_ α4β1 integrin 8.3 nM). Consistently, DS70 reduced Jurkat cell adhesion to VCAM-1 (IC_50_ 5.04 nM) or FN (IC_50_ 4.3 nM). Furthermore, DS70 antagonized VCAM-1-mediated phosphorylation of ERK 1/2 in Jurkat E6.1 cells. Finally, the effects of topical treatment with DS70 on a guinea pig model of allergic conjunctivitis were evaluated, following direct administration in the conjunctival fornix. DS70 dose-dependently reduced clinical aspects of allergic conjunctivitis, conjunctival mast cell and eosinophil infiltration, α4 integrin expression, and levels of mRNAs for IL-1β, IL-8 (CXCL8), CCL5, and CCL11, thus representing an alternative to antihistamines and mast cell-stabilizing agents.

### Addressing Integrins with Agonist Ligands

Even though most of the efforts have been directed to the development of integrin antagonists, the discovery of agonists could represent a new perspective [1]. In recent years, some agonist molecules have been disclosed, targeting different types of integrins. Celik and co-workers [46] studied the capacity of leukadherin-1 to act as an agonist for αMβ2, and thus be able to reduce neutrophil migration and inflammatory response. Using the structure of the well-known antagonist TBC3486, Dixon and co-workers [47] designed and tested in vitro a new α4β1 agonist, with the aim of increase cell retention and so improve progenitor cell therapy. Because previous works have shown that chemotherapy can affect more metastatic melanomas when cells are adherent to ECM instead of suspended. Schwartz and co-workers [48] tested in vivo the chemotherapeutic drug combined or not with contortrostatin, a disintegrin from snake venom, and proved that stimulate integrin may help the efficacy of chemotherapy and help to reduce tumor growth. As the interaction between αLβ2 and ICAM-1 plays an important role in the immune responses and leukocytes adhesion, this integrin has been the target of several studies. In particular, small molecule agonists were developed and tested [49] to demonstrate that stimulation of αLβ2 integrin facilitates cell signaling.

**Figure 4:**
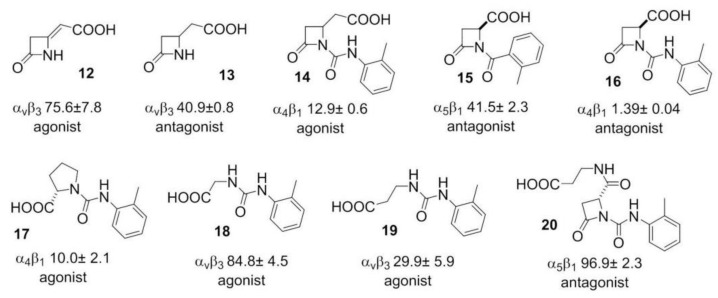
Examples of β-lactam based agonist or antagonist integrin ligands utilized for Structure-Activity Relationships (SAR) and modeling analysis of agonism vs. antagonism; the affinities (nM) for the specified integrins are also shown.

More recently, β-lactam-based ligands were designed for investigating the structural determinants of agonist vs. antagonist activity [50,51]. The new ligands (Figure 4) showed nanomolar affinity and selectivity for integrins ανβ3, α5β1, or α4β1, and agonist (**12**, **14**, **17**–**19**) or antagonist (**13**, **15**, **16**, **20**) activities on integrin-mediated cell adhesion. The best agonist molecules induced significant adhesion of SK-MEL-24 cells and Saos-2 cells as a valuable model for osteoblast adhesion. The latter result could lead to the development of new agents to improve cellular osseointegration and bone regeneration. Molecular modeling and docking studies on ανβ3 or α5β1 integrin supported the notion that ligand carboxylate fixing to the metal ion-dependent adhesion site in the β-subunit can be sufficient for binding the receptors, while the aryl side chains play a role in determining the selectivity as well as agonism versus antagonism [52].

## 5. Integrin-Targeted Peptide Conjugates

**Figure 5:**
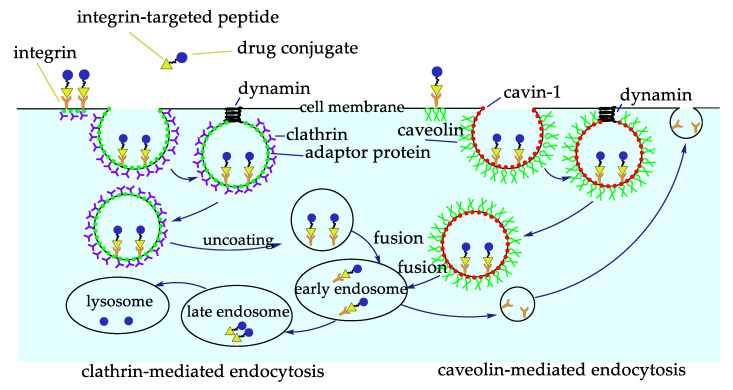
Integrin-targeted internalization of the Arg–Gly–Asp (RGD) peptide-drug conjugates via clathrin or caveolin mediated endocytosis.

The scarce bioavailability of most anticancer drugs represents a significant obstacle to their efficacy. Besides, most anticancer drugs also show a certain toxicity towards healthy cells. These problems can be solved by coupling the drugs to a carrier to improve its targeted delivery and internalization. There are several advantages for the construction of a carrier system, one of them is the ease of these systems to be internalized via receptor-mediated endocytosis, and thus be able to arrive in regions away from blood vessels, the ones previously impossible to reach [53]. Active and inactive integrins undergo a clathrin-mediated or caveolin-mediated endocytosis-recycling cycle, involved in different processes, such as cell migration, by detaching integrins from the extracellular matrix, integrin recycling and activation of different receptors (Figure 5) [54]. Considering this, interest has focused on the construction of RGD conjugates with anticancer drugs, diagnostic probes, NPs, or nanocarriers for cancer therapy or imaging [2,55,56]. All of these drugs are designed to be more effective and reduce collateral damages.

Among these approaches, antibody-drug conjugates (ADCs) have met great success. However, they often suffer from limitations ascribable to their dimensions and their possible immunogenicity [57]. Small molecule-drug conjugates (SMDCs) are an alternative approach to ADCs, as they are usually designed to include a drug and a targeting ligand linked by a spacer [58]. To develop tumor-targeting conjugates, it is suggested to introduce a cleavable bridge, which must be stable in human fluids, while being able to cleave and release the therapeutic payload after penetrating the tumor cells. Most SMDCs are internalized by the cell through receptor-mediated endocytosis; the internalized conjugate is then transferred to the early endosomes or in the lysosomes. As ADCs and SMDCs have been duly reviewed elsewhere [2,56,58,59], these topics will be not analyzed in detail in this review.

## 6. Integrin-Targeted NPs

Most NPs used in biomedicine are inorganic, organic, or mixed, particles with dimensions comprised between 1 and 100 nm. Therapeutic NPs can be coupled to diagnostic probes, giving multimodal agents. In particular, the combination of NPs and RGD peptides has been widely explored in cancer and cardiovascular diseases [2,56].

Nanoparticle delivery systems can be exploited to passively target the tumor and able to penetrate cancer cells, taking advantage of the enhanced permeation and retention (EPR) effect. NPs functionalized with properly disposed of peptide ligands onto the surface can promote the multivalent targeting of integrin receptors. The cargo-bearing NPs approach the receptor-embedded plasma membrane. The ligands bind the recognition domain of the receptors and trigger receptors clustering, and the cargo is internalized for subsequent intracellular trafficking. Binding NP to integrins can activate signaling pathways and subsequently affect cell proliferation, differentiation, or migration. Integrins synergize with other cell surface receptors, such as receptor protein tyrosine kinases, to activate signaling via ERK1/2 cascade [60].

NPs can be adsorbed onto plasma proteins in the bloodstream, then mononuclear phagocyte system may recognize and remove them from circulation. NPs surfaces can be coated with polyethyleneglycols (PEGs), to confer stealth properties with respect to non-specific uptake by the reticuloendothelial system (RES). Pegylation is a widely adopted strategy to increase circulating time in vivo, mostly due to the ability to evading macrophage-mediated uptake and removal from the systemic circulation. In addition, PEG prevents other molecules to bind by steric effects, as well as non-specific binding to proteins and cells [61].

Many protocols have been proposed for NP functionalization. The coupling strategy to be adopted depends on the stability of the NPs, the functional groups, the bioconjugation conditions, and the biomolecule to attach. Besides, depending on the conjugated biomolecule, it is important to control the orientation, so that the biomolecule remains active once conjugated to the NP. Biofunctionalization can be achieved by covalent coupling, including conjugate maleimide to thiols, or the azide-alkyne cycloaddition (CuAAC) reaction catalyzed by copper (I) to introduce functional groups, PEG or proteins. Alternatively, non-covalently physical interactions are also described but compared to covalent functionalization, they show some weaknesses, such as scarce stability, and the unreproducible and uncontrollable amount and orientation of the functionalization [62].

### 6.1. Integrin-Targeted Organic NPs

#### 6.1.1. Liposomes

**Figure 6:**
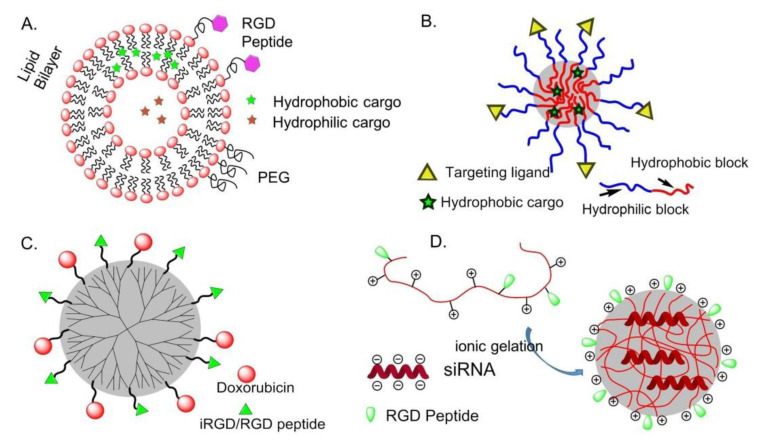
Integrin-targeted organic nanoparticles (NPs). (**A**) liposomes; (**B**) polymeric micelles; (**C**) dendrimers; (**D**) chitosan NPs.

Compared with other drug delivery systems, liposomes loaded with chemotherapeutic agents are supposed to prolong the circulation time in blood, increase the bioavailability and cell uptake [63]. Immuno-liposomes can be utilized to deliver cargos to targeted tissues, avoiding toxicity and side effects to normal tissues, and bio-responsive liposomes are designed for specific tissue targeting and controllable drug delivery [64]. Hydrophobic and hydrophilic molecules are inserted into the lipid bilayer or aqueous compartments, respectively (Figure 6A). This is particularly important for drugs such as paclitaxel (PTX), which is known to induce severe side effects and is practically insoluble in water. 

To drive the liposomes towards the desired cells (e.g., cancer cells), preformed liposomes can be modified by conjugation with integrin ligands [65]. More often, integrin targeting liposomes have been prepared using phospholipids carrying integrin ligands [66]. To improve targeting of tumor vasculature, in 2010, Meng et al. prepared PTX-loaded liposomes, carrying RGD peptide and the ATWLPPR sequence, which is the ligand of the VEGF receptor neuropilin-1 (NRP-1). The bimodal liposomes enabled a greater internalization of PTX than either each of two single-targeted liposomes [67].

#### 6.1.2. Polymeric Micelles (PM)

Amphiphilic block or graft copolymers tend to self-assemble in aqueous media giving globular colloidal polymeric micelles (PM). Their core-shell architecture consents to load lipophilic antitumor agents into the hydrophobic core and the outer hydrophilic shell allows NPs to be stable in aqueous solution (Figure 6B). Generally, the copolymers are composed by hydrophobic materials that easily undergo hydrolytic or enzymatic degradation, such as PCL (poly (ε-caprolactone)), PLA (poly (lactic acid)), PLGA (poly(lactic-co-glycolic acid)), or temperature or pH variations, whereas PEG is commonly utilized for the hydrophilic segment [68]. 

In 2004, Gao and co-workers first prepared micelles composed of a PEG-PCL block copolymer ending by a maleimide, to which a thiolated-cRGDfK was conjugated. These micelles were able to deliver into SLK tumor cells [69]. The same authors subsequently prepared RGD-functionalized PEG-PLA micelles loaded with Dox and superparamagnetic iron oxide NPs (SPIONs), for combining therapy and imaging (ultrasensitive MRI) [70]. In these examples, the payload is released upon structure degradation. In some cases, the drug is covalently conjugated to the block copolymer by a hydrazone bond or an amide bond [71].

RGD-functionalized biocompatible synthetic polymers such as PEG, PLA, PLGA, PLL (poly (lactic lysine)), or natural polymers, chitosan, albumin, collagen, have been utilized to prepare integrin targeting biodegradable polymeric NPs. Drugs such as Paclitaxel, Mitoxantrone, and Fluorouracil can be incorporated in micelles without chemical modification or with conjugation to the polymers. Then, it can be released in a controlled manner by diffusion through the polymer matrix, by polymer degradation [72].

#### 6.1.3. Dendrimers

In 2009, Waite et al. found that conjugating cyclic RGD to a poly(amidoamine) (PAMAM) dendrimer enhanced the penetration and delivery of short-interfering RNA (siRNA) through tumors in a manner that depended on the targeting ligand density [73].

In 2014, an effective anti-tumor drug delivery system PPCD (PEG-PAMAM-cis-aconityl-DOX) was prepared by covalently bonding or simply mixing the tumor penetrating peptide iRGD (internalizing-RGD, CRGDK/RGPD/EC) and the PAMAM dendrimer. Experiments have shown that it can increase the permeability of the tumor blood vessel and the accumulation of drugs in the tumor tissue [74] (Figure 6C).

#### 6.1.4. Chitosan

A positively charged chitosan NP is a talented siRNA delivery vehicle because of its advantage in cellular membranes transportation and endocytosis (Figure 6D). In 2010, Sood et al. conjugate RGD peptide to chitosan NP by thiolation reaction for targeted siRNA delivery. This strategy significantly enhanced siRNA delivery to tumor tissues and vasculature, binding efficiency on ανβ3 integrin-expressing tumor cells, and therapeutic efficacy of gene silencing [75].

#### 6.1.5. Other Organic NPs

Doxorubicin-loaded human serum albumin NPs conjugated with an antibody targeting ανβ3-positive M21 melanoma cells exhibited enhanced cytotoxicity as compared to free doxorubicin [76]. In 2013, splice-switching oligonucleotides (SSOs) was utilized to bond RGD peptide to serum albumin to prepare a nanoconjugate with a diameter of 13 nm. Because of the small size of nanoparticle and grafting of the tumor-targeting peptide, the ability to penetrate receptor-specific cells was 61 times higher than the control. Compared with other siRNA delivery vehicles, this nanoconjugate exhibits the advantages of high loading rate, increment tumor specificity, strong tumor permeability, and high therapeutic oligonucleotide activity [77].

Poly (cystaminebisacrylamide-diaminohexane) [poly (CBA-DAH)] (CD) is a biodegradable and low-toxic polymer, that was bond to a tumor homing peptide c[RGDfC] by biofunctionalized PEG, c[RGDfC]-PEG-CD shielded on adenovirus (Ad) for reducing cytotoxicity and improving transduction efficiency. This oncolytic Ad expressing short hairpin RNA (shRNA) against interleukin-8 (IL-8) mRNA, after Ad/CD-PEG500-RGD was introduced in HT1080 cells. The expression of IL-8 and VEGF was inhibited and then induce apoptosis [78].

### 6.2. Integrin-Targeted Inorganic NPs and QDs

Inorganic NPs of gold, silver semiconductors, magnetic compounds, alloys, silica, etc., display unusual size-dependent optical and/or magnetic properties, drastically different from those of their bulk materials, exploitable for detection and imaging, and the targeting of multifunctional therapeutics [79].

#### 6.2.1. Silica NPs

Silica NPs as tools to develop targeting probes and drug delivery systems have several advantages over other nanomaterial and self-organized systems. Indeed, silica is photophysically inert, is an intrinsically non-toxic material, and there are many synthetic approaches available to tune these nanosystems in terms of size and functionalization. The luminescence emission of these systems depends on the doping dye so that a large variety of emission properties can be achieved by just choosing the right doping dye(s). The inclusion of dye molecules in rigid matrix-like silica often increases the quantum yield of the dyes and also their photostability, because of the rigidification of dye structure and the protection towards quenching molecules present in the environment. These last two features are of prominent importance to univocally assign the recorded fluorescent signal to the presence of the NPs and to control the local concentration of the cytotoxic compound during the recognition event toward the targeted receptor. 

In 2018, Jia et al. generated bifunctional 40 nm-sized silica NPs coated with controlled amounts of the peptides cRGD and ATWLPPR (neuropilin-1 (NRP1), a co-receptor of VEGFR2) and studied their affinity, selectivity and biological activity in HUVECs (Human Umbilical Vein Endothelial Cells). The results supported evidence for a complex cross-talk generated by the binding of the heteromultivalent NPs with ανβ3-integrin and with NRP1. In particular, the NPs exerted dose-dependent pro-survival activity. This study demonstrated the difficulties in designing targeted silica-based NPs for antiangiogenic therapies and the possible risks posed by undesirable side effects [80].

In 2020, Juthani et al. introduced ultrasmall fluorescent core-shell silica NPs, Cornell prime dots (C’ dots), were functionalized with cRGD peptide, and PET (Positron Emission Tomography) labels (124I, 89Zr) to investigate the utility of dual-modality cRGD-C’ dots for enhancing accumulation, distribution, and retention (ADR) in a genetically engineered mouse model of glioblastoma (mGBM). The results showed improvements in brain tumor delivery and penetration, as well as enhancement in the ADR, were observed following administration of integrin-targeted C’ dots, as compared with a nontargeted control [81].

**Figure 7:**
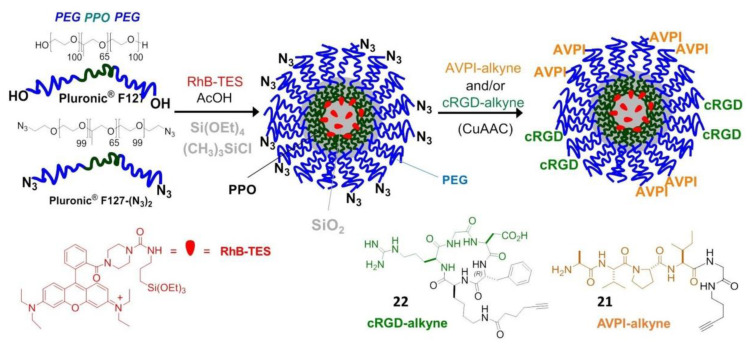
Preparation of AVPI/cRGD-NPs from micelles composed of PF127 (PEG_100_–PPO_65_–PEG_100_) and PF127-(N_3_)_2_, followed by polymerization of the silica core to embed the dye RhB. Peptide functionalization is achieved by click chemistry.

Very recently, peptide-functionalized silica NPs mimicking the proapoptotic protein Smac/DIABLO were prepared and validated in vitro. These NPs were constituted by a fluorescent silica core doped with rhodamine B (RhB), coated with a PEG shell, and carrying the AVPI peptide and/or a tumor-homing cRGD peptide. The preparation started from micellar NPs composed of the tri-block surfactant copolymer Pluronic^®^ F127 (PF127) (polyethylene glycol-polypropylene oxide -polyethylene glycol, PEG_100_–PPO_65_–PEG_100_) followed by condensation of the silica core (Figure 7). 

For the purpose of peptide conjugation, the NPs were prepared from a mixture of PF127 and its diazide derivative PF127-(N_3_)_2_, and submitted to click chemistry with AVPI-alkyne, containing the pro-apoptotic AVPI N-terminus of Smac/DIABLO (**21**), or the integrin-targeting cRGD-alkyne (**22**), or a 1:1 mixture of both. At low μM concentration, the bifunctional AVPI/RGD-NPs showed superior toxicity towards A549, U373, and HeLa cancer cells and modest toxicity towards other integrin-expressing cells, correlated with integrin-mediated cell uptake and consequent highly increased levels of apoptotic activity, without perturbing cells not expressing the α5 integrin subunit [82].

#### 6.2.2. Magnetic NPs

In 2009, the Ruoslahti group reported a cyclic peptide combining the tumor-homing RGD sequence with a tissue penetration motif. The homing sequence directs the peptide to the tumor vascular endothelium, while the tissue penetration motif, once activated by a protease, binds to a different receptor (neuropilin-1), which mediates extravasation and tissue penetration. As a proof of concept, iRGD peptide-linked iron oxide nanoworms could be detected by MRI throughout a tumor once injected in vivo to mice [83]. In 2011, the same group combined two different peptides with the magnetic nanoworms to image and treat mice with glioblastoma, one of the most difficult tumors to treat. While the CGKRG peptide targets the NPs to tumor vascular cells and into their mitochondria, the other peptide acts as a pro-apoptotic drug. By co-injecting these NPs with iRGD, most of the tumors were eradicated or their development delayed in two glioblastoma mouse models [84].

**Figure 8:**
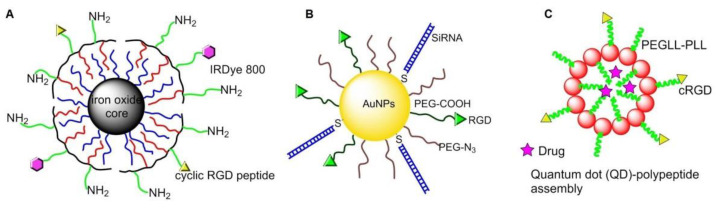
Integrin-targeted inorganic NPs and quantum dots (QDs): (**A**) magnetic NPs; (**B**) gold NPs; (**C**) QDs.

PEGylated copolymer-coated iron oxide NPs conjugated with an RGD-containing cyclic peptide c(RGDyK) was administrated in a mouse model for targeting αvβ3 integrins. Successful tumor homing was perceived in a subcutaneous U87MG glioblastoma xenograft model by magnetic resonance imaging (MRI) [85] (Figure 8A). Similar integrin specific binding was achieved on HUVEC cells by using paramagnetic liposomes conjugated with the cyclic RGD peptide [86].

#### 6.2.3. Gold NPs

In 2013, Conde et al. conjugated RGD peptide and thiolated siRNA to PEG-modified Au NPs, making it possible to deliver siRNA to tumor cells and effectively silence target oncogenes, then down-regulate c-Myc oncogene and inhibit tumor growth, as well as prolong the survival time of mice with lung cancer [87] (Figure 8B). In 2015, Zhang and coworkers bonded c[RGDfK] to PEG-entrapped Au-NPs (Au DENPs-RGD) as a nanoprobe. After intravenous injection into tumor-bearing mice, the NPs accumulated in tumor and were detected by CT imaging [88].

#### 6.2.4. Quantum Dots (QDs)

In 2010, Atmaja et al. reported a tunable QD-polypeptide assembly system by self-assembly of QDs and c(RGD)-PEGLL-PLL (Poly (diethylene glycol-L-lysine)-poly (L-lysine)) diblock (Figure 8C). This system entered into the cells by endocytosis, and the cargos were released due to loss of self-assembled electrostatic interactions, allowing simultaneous molecular imaging and drug delivery [89].

In 2015, Hu et al. modified the peptide RGD and peptide bombesin (BBN) onto the surface of QDs and then radiolabeled with 4-nitrophenyl-2-^18^F-fluoropropionate to synthesize PET/NIRF probe ^18^FFP-QD-RGD-BBN. By studying PC-3 cells and prostate tumor-bearing mice with dual-mode PET and NIRF imaging, the biodistribution and tumor targeting of QDs could be determined, and tissue penetration in optical imaging was also improved [90]. 

## 7. Surfaces and Materials Functionalized with Peptidic Integrin Ligands

Peptide-functionalized surfaces are important both for studying integrin-mediated cell adhesion, growth, spreading and differentiation, and in biomedicine, for implant materials and tissue engineering [91]. ECM derived peptides or protein fragments that specifically engage integrins were used to functionalize implants and orthopedic biomaterials, to upregulate the formation of osteoblasts, enhance cell adhesion, proliferation, and differentiation [92]. Different approaches have been developed to promote material surface bioactivation [93,94]. The mere absorption of the ligands on the surfaces leads to unpredictable, nonspecific, and potentially unstable interactions both with the cells and the material surface. Much better results have been obtained with mechanically and chemically stable linkers. To introduce those linkers without affecting functions of immobilized-peptides and without interfering other remaining amino acids, a series of chemical reactions have attracted much attention: the palladium-catalyzed or copper-co-catalyzed Suzuki–Miyaura, Sonogashira, and Mizoroki–Heck reaction; the cycloaddition reactions, such as Cu-catalyzed azide-alkyne cycloaddition, Diels–Alder reaction, and Huisgen cycloaddition; the olefin metathesis reaction promoted by Grubbs or Hoveyda–Grubbs catalysts; the Staudinger ligation, utilized in chemical biology for the synthesis of fluorescently labeled nucleosides [95]. In this context, very recently De Marco et al. developed an expedient synthesis of hybrid peptides containing imidazoline-2-one scaffolds [96]. The rings were equipped with functional groups capable of selective reactions without perturbing the rest of the structure, for example, the Heck reaction on the allyl group. Potential applications might include the glycosylation, prenylation, PEGylation, biotinylation, or attachment to solid surfaces, SAMs, proteins, or the conjugation with fluorophores or antibodies. Intriguingly, these pseudo-proline heterocycles tend to favor unusual conformations, including the ε-turn, a rare secondary structure characterized by an 11-membered 2→4 H-bond going opposite respect to the classic turns. 

The interaction between cells and the materials is based on several parameters, among which the density of integrin ligands anchored on the surface of the materials is the most important one. Besides, the spatial distribution of the adhered moieties on the surface is also an indispensable factor, which affects the accessibility of the peptide to the cells. By conjugating the integrin ligand to the surface with different density and spatial arrangement, important insights have been provided into how the organization of the ligands can influence integrin clustering, FA formation, and subsequent adhesion and spreading of cells. In particular, it has been discovered that there is a critical lateral spacing of approximately 60–70 nm between integrin ligands. When the spacing is out of range, integrin clustering and FA formation are hindered, thereby limiting cell attachment and diffusion [97]. The limitations of spatial distance and peptide arrangement may be related to physiology because ordered structures happen in the native ECM.

### 7.1. Self-Assembled Monolayers (SAMs)

SAMs of GRGDS peptides on gold were fabricated by using thiolated PEG linkers containing 3 to 6 monomers. An increase in the length of the polyethylene glycol chains resulted in a decrease in Swiss 3T3 fibroblast cell adhesion and spreading, especially for lowest ligand density [98]. SAMs of cyclic RGD (1 mol.%) were further used to investigate the dynamics of cell migration in the presence of a linear RGD antagonist. In a definite concentration range, cell migration speed increased upon increasing the concentration of the antagonist [99].

SAMs of RGD peptides were prepared on silicon surfaces with peptide spacing ranging from nanometers to micrometers. The silicon materials were modified with undecenoic acid and mixtures of 1-amino hexa(ethylene oxide) monomethyl ether and 1-amino hexa(ethylene oxide) in various ratios. The alcohol terminus of the hexa(ethylene oxide) was activated to a succinimide ester to consent the pentapeptide RGD to be coupled to the surface. Endothelial cells adhered to and spread on surfaces independently of RGD spacing. However, the formation of FAs was particularly sensitive to the ligand spacing and the optimal spacing for RGD was found to be 44 nm [100]. 

### 7.2. Interfaces for Studying of Cell Adhesion, Spreading, and Differentiation

To promote cell binding, two domain-peptide ligands were simply absorbed onto gold. One domain is an anchoring domain for AuΦ3 gold binding, and the other is an IKVAV or RGD motif present in ECM proteins as a cell-binding domain. Compared with the sequence containing only the cell-binding domain, the sequence with the anchoring domain had higher adsorption strength, which can induce cell polarization and larger mature FA area. These correspond to the high forces exerted on the interface and they enhance cell interaction with the material [101].

In 2009, Garcia et al. prepared supported lipid monolayers (SLMs) directly on octadecyl trichlorosilane-coated substrates. These surfaces were utilized to study the adhesion of hematopoietic progenitor cell lines to a peptide derived from FN [102]. In 2001, Sackmann and co-workers prepared artificial membrane giant vesicles which incorporated 1–10 mol.% of c[RGDfK]-lipopeptide. After seeding, endothelial cells remained adherent and spread on RGD-SLM, while cells remained round on control SLMs [103].

Supported lipid bilayers (SLBs) are biological interfaces mimicking cell membrane, with easily tunable characteristics. The head group of the lipids can be functionalized with integrin-binding peptides. In 2017, in Jonkheijm’s group, biotinylated 1, 2-dioleoyl-*sn*-glycero-3-phosphocholine (biot-DOPC) and 1,2-dipalmitoyl-sn-glycero-3-phosphocholine (DPPC) were utilized to obtain ligand-mobile SLBs, which was functionalized with linear biotinylated-RGD thanks to the intermediacy of neutravidin. The resulting RGD-mobile SLBs were employed to study short term cell adhesion and longer-term cell differentiation of human mesenchymal stem cells (hMSCs), and the result showed that cell adhesion and differentiation positively correlated to ligand density and mobility [104].

### 7.3. Application in Regenerative Medicine and Tissue Engineering

In 2013, Rechenmacher et al. utilized click chemistry to immobilize peptidomimetics of α5β1- or ανβ3-selective RGD peptides on Ti-based materials via phosphonic acid-containing anchoring units, and the resulting surfaces promoted the selective binding of α5β1- or αvβ3-expressing fibroblasts [105]. In 2015, Fraioli et al. reported that these Ti-peptide surfaces also allowed the adhesion, proliferation, and differentiation of OB-like cells, hence representing prototypes for implant materials with osteoinductive properties [106].

Track-etched (TE) microporous membranes of polyethylene terephthalate were grafted with GRGDS peptide or peptidomimetic ligands of ανβ3 integrin, via trifluorotriazine activation. These devices were described by Rémy and his coworkers in 2013 and showed improved adhesion of human endothelial cells under shear stress mimicking arterial conditions. The optimal number of peptide molecules grafted on the surface was about 50 pmol/cm^2^ [107], whereas cells were not observed on the surface of non-grafted PET.

Aiming at improving the adhesion of bone-forming osteoblasts at the surface of implants for regenerative medicine, in 1999, Kessler et al. proposed a method for the coating of the implants using integrin-specific ligands. Osteoblasts were found to be effectively bound to the material poly(methyl methacrylate) (PMMA), which anchored the cRGD peptide with N-succinylcysteamide or a 3-sulfanylpropionic acid linkers [108].

The functionalization of collagen scaffolds with RGD ligands that support cellular attachment has been extensively studied by Schussler and his coworkers in 2009, for cardiac tissue engineering in the treatment of diseased myocardium or cardiac malformations [109]. In 2012, in the Kilian group, SAMs of cRGD ligands were obtained by conjugating the c[RGDfC]/GRGDSC peptides to Au-SAMs using thiolated tri-(ethylene glycol) linkers. The differentiation of MSCs was affected by the affinity and density of an immobilized ligand for the integrin receptors. As a result, MSCs on monolayers of c[RGDfC]-SAM-Au showed increased expression of osteogenic markers, while cells on monolayers of GRGDSC-SAM-Au expressed early markers of myogenesis at a high density and neurogenesis at a low density of the ligand [110].

### 7.4. Fabrication Methods of Integrin Ligand Immobilized Nanostructured Surfaces

The random presence of cell adhesive ligands on a material surface alone is not sufficient to elicit a full cell adhesion response, being the nanoscale distribution of these ligands on the surface also critically important. Indeed, to promote the formation of FAs, the integrin receptors must be clustered within the cell membrane. Integrin clustering can be favored by culturing cells on surfaces functionalized with multivalent ligands. A variety of fabrication methods have been developed to control the nanoscale presentation of integrin-binding ligands on biomaterial substrates, including blending, electron beam patterns, photolithography, and nanolithography, electrospinning, 3D printing, etc. [111]. 

#### 7.4.1. Blending Strategy for Surfaces Functionalization

In 2008, Becker and Simon fabricated fibroin and synthetic RGD-containing spidroin (RGD-spidroin) on glass coverslips in different proportions of RGD-spidroin from 0% to 70%. The higher ratio of RGD-spidroin is related to the high content of the β-sheet, which has a positive correlation with film stability and cell adhesion, but an insignificantly negative correlation with differentiation. It was also found that the optimal proportion of RGD-spidroin was 10% for film stability, osteoblast adhesion, and differentiation [112]. 

In 2014, Yang et al. prepared bio-fibers by blending RGD-containing peptides functionalized mussel adhesion protein (MAP-RGD) into silk fibroin (SF). It was determined that MAP-RGD-SF not only improved the attachment, proliferation, and spread of mammalian cells but also promoted the adhesion of carbohydrates and proteins. Compared with SF, MAP-RGD-SF had high hydrophilicity, biodegradability, and wettability, which made it have greater potential in the application of tissue engineering and regeneration medicine [113]. In 2018, Janani et al. prepared a 3D porous silk scaffold by blending mulberry silk fibroin protein and RGD-containing non-mulberry silk fibroin protein to develop bioartificial liver constructs. It was found that the blend scaffold increased the density of hepatocyte clusters and retained liver-specific functions for 3 weeks [114].

**Figure 9:**
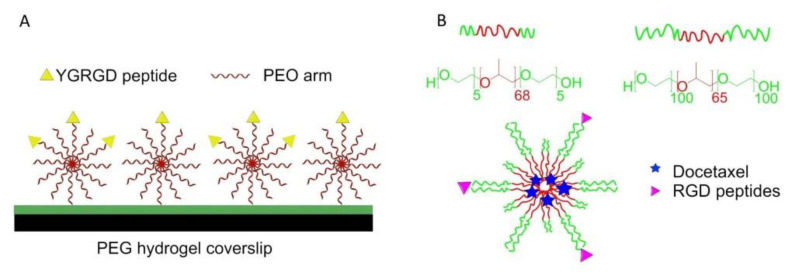
Blending strategy for surface functionalization. (**A**) Star PEO was blended on the surface. The surface density of star PEO and special distribution of RGD peptides can be controlled. (**B**) Preparation of cRGD-conjugated tumor-targeting drug delivery platform by blending of L121 and F127 and encapsulating docetaxel.

In 2000, Maheshwari et al. reported a Star-shaped polymer containing many PEO arms. The YGRGD attached star-shaped PEO was blended with the unfunctionalized star-shaped polymer covalently tethered on the PEG hydrogel-modified coverslip to independently control the peptide density and spatial distribution of the surface. It was found that NR6 fibroblast cells could only migrate on the YGRGD peptide adhered surfaces (Figure 9A) [115].

A cRGD-conjugated micellar system was prepared by blending pluronics L121 and F127 to increase docetaxel-loading capacity and particle stability (>1 week). It was also found that the enhancement of cellular uptake improved anticancer activity against U87MG cancer cells, and tumor-targeting accumulation of blending micellar systems in vivo (Figure 9B) [116]. 

#### 7.4.2. Electron Beam Fabricated Patterns

In 2007, Rundqvist et al. proposed an electron beam as a high-fidelity approach for surface coating with proteins [117]. A silicon substrate was coated with protein fibronectin, which was then inactivated by an electron beam. The area exposed to the electron beam lost the ability of Ab binding and cells spreading. The inactivation was dependent on the dose of electron and accelerating voltage. In contrast to the ablation observed in other organic thin films, this patterning approach allowed local inactivation, and the level of protein patterning could be controlled to a single molecule.

**Figure 10:**
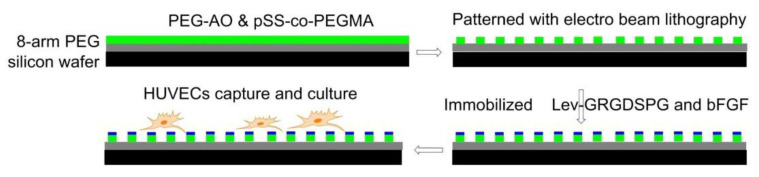
Electron beam lithography. Before immobilizing Lev-GRGDSPG and bFGF on the surface, pSS-co-PEGMA and PEG-AO were coated and cross-linked on passivated silicon wafers. Then HUVEC cells were cultured on the surface.

Using electron-beam lithography, in 2012, Maynard [118] functionalized substrates with both Lev-GRGDSPG peptide and basic fibroblast growth factor (bFGF). First, 8-arm PEG-OH was coated on a silicon wafer and passivated by thermal annealing, then a 1:1 blend of PEG-AO (8-arm aminooxy-terminated PEG) and pSS-co-PEGMA (Poly(styrene-4-sulfonate-co-poly(ethylene glycol) methacrylate)) was patterned by electron beam lithography. Finally, GRGDSPG and bFGF were immobilized on the surface. The presence of RGD promoted cell adhesion and the formation of FAs. Compared with the surface containing only RGD, the surface containing both RGD and bFGF increased the cell area of HUVEC (Figure 10).

#### 7.4.3. Photolithography Strategy for Surfaces Functionalization

Using photolithography technology, polydimethylsiloxane (PDMS) microgrooves were prepared in 2017 by Kim and his coworkers and then immobilized by filamentous phages, which were genetically functionalized with RGD peptide. The presence of RGD-phages in PDMS-microgrooves enhanced adhesion, proliferation, and orientation of H9c2 cardiomyocyte, which has application prospects in stent coating materials [119].

In 2017, in the Bilem group, RGD peptide and bone morphogenetic protein (BMP) mimetic peptide were immobilized on glass surfaces by geometrically controlled photolithography patterning. hMSCs osteogenic differentiation-related to the spatial distribution of two different peptides and shape of peptide micropatterns. Compared with the BMP-2 functionalized surface only, the RGD/BMP-2 modified surface could significantly promote hMSC osteogenesis [120]. In 2018, the same group described that spatial patterning surfaces of RGD-TAMRA did not change the stemness character of hMSCs, but the presentation of BMP-2-FITC peptide on material surfaces promoted the differentiation of hMSCs into osteoblasts. Triangular and square peptide patterns with an aspect ratio of 1 and 0.7 significantly enhanced hMSCs osteogenesis [121].

**Figure 11:**
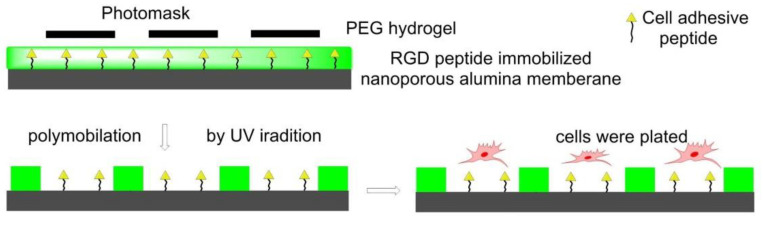
Photolithography strategy for surface functionalization. The photoinitiator-containing gel precursor solution was added on RGD peptides immobilized nanoporous alumina membrane to form a thin layer. Then it was covered by photomask and irradiated by UV light to get polymerization. Fibroblasts cells were seeded in the nanowells.

In 2011, Using photolithography, RGD-immobilized nanoporous alumina membrane was immobilized with PEG hydrogel micropatterns to construct microwells with different sizes in Koh’s lab. Fibroblasts adhered and proliferated in RGD-containing microwells instead of PEG hydrogel walls to form a cellular micropattern. The formation of filopodia and its penetration into nanopores was also observed (Figure 11) [122].

#### 7.4.4. Nanolithography Strategy for Surfaces Functionalization

A substrate-patterning strategy was based on the self-assembly of diblock copolymer micelles. After the assembly of the Au-dot-containing micelles, the polymer was entirely removed by a gas plasma treatment, which resulted in extended and highly regular Au nanodots, deposited into a nearly perfect hexagonal pattern on substrates such as glass. c[RGDfX] peptides were bound to gold NPs structured surfaces via polyproline, polyethylene glycol, or aminohexanoic acid-containing spacers of different lengths. Changes of the ligand’s spacer chemistry and length reveal significant differences in cell adhesion activation and FA formation. An increase in spacer length resulted in an increase in FA density in fibroblasts as well as an increase in the average cell area. Longer and more hydrophilic spacers induced more stress fiber formation. Short hydrophobic spacers induced the slowest cellular spreading rate; and for all spacers, increasing their length led to an increase in the rate of cell spreading. Polyproline-based peptides demonstrated improved cell adhesion kinetics and FA formation compared with common aminohexanoic acid or polyethylene glycol spacers, correlated to the much higher extension and rigidity of the former. Binding activity was additionally improved, inducing a higher and faster cell spreading, by ligand dimerization, obtained by joining two cRGD peptides with a lysine branch [123].

In 2014, in Cavalcanti–Adam’s lab, Gold NPs were deposited on β-type Ti-40Nb alloy discs by block copolymer micelle nanolithography and were subsequently functionalized with thiolated cRGD ligands in defined patterns. When seeded with human MSC, these nanostructured discs showed reduced cell heterogeneity, while their adhesion, in terms of cell size and FA formation, was optimized [124].

**Figure 12:**
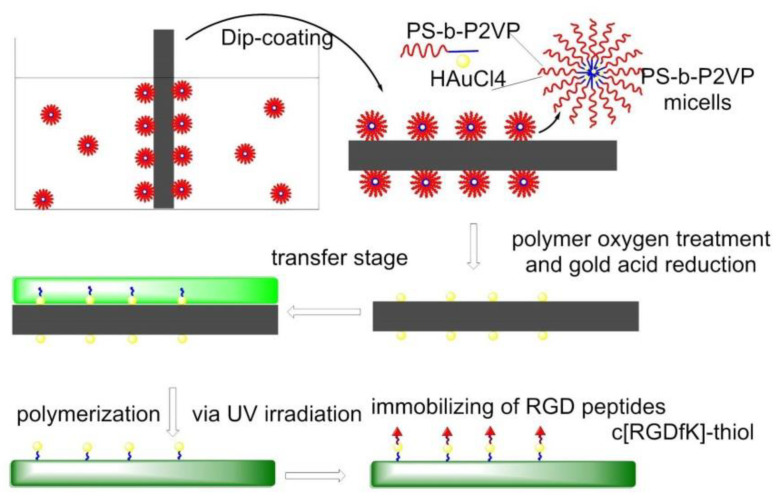
Nanolithography surfaces functionalization. PS-b-P2VP micelles were dip-coated on glass, forming Au-nanopatterned surfaces. c[RGDfK]-thiol was anchored on gold before cell culture.

In 2013, gold nanopatterns on persistently non-fouling PEG hydrogels were prepared by Wang et al. via block copolymer micelle nanolithography plus transfer nanolithography. Specifically, micelles of amphiphilic block copolymer polystyrene-block-poly(2-vinyl pyridine) (PS-b-P2VP) were reacted with HAuCl_4_ to give Au-enriched micellar cores. The process of preparation of a gold nanopattern on glass exploited dip-coating and oxygen plasma, then the Au nanopatterns were transferred from glass to a PEG hydrogel, which was formed via photopolymerization of PEG-diacrylate (DA), and then RGD motifs were grafted onto the gold nanodots to eventually obtain an RGD nanopattern on the surface of a PEG hydrogel. The behaviors of MSCs on diverse pattern nanospacings were examined under a full level of serum, confirming less spreading in the case of nanospacings larger than the critical 70 nm. Osteogenic and adipogenic inductions resulted in higher differentiation extents on patterns of large nanospacings than of small nanospacings (Figure 12) [125].

In 2013, nanodots were fabricated on silicon surfaces by Cheng et al. and modified with RGD peptide by combining nanoimprint lithography technology with surface modification techniques. Square arrays of nanodots were transferred from a silicon mold onto silicon substrates via a polymer mask. These patterns were functionalized with a 3-aminopropyldimethylethoxysilane (APDMS) layer subsequently grafted with a cysteine-modified GRGDSPC peptide through a 3-succinimidyl-3-maleimidopropionate (SMP), a heterobifunctional cross-linker. After polymer mask removal, the nonpatterned background was passivated with a cell-repellent PEO-silane layer. These systems were utilized for studies in human mesenchymal stem cell adhesion, differentiation, and induction of FA [126].

#### 7.4.5. Electrospinning

**Figure 13:**
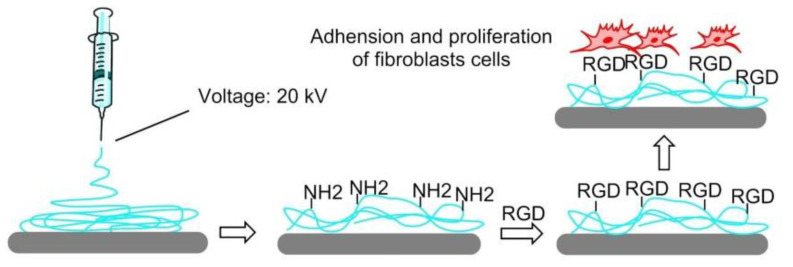
The electrospin strategy for surface functionalization. A mixture of PLGA (poly(lactic-co-glycolic acid)) and PLGA-b-PEI-NH_2_ in DMF/THF was electrospun to produce nanofiber with free NH_2_-group, which can be used to conjugate RGD peptides. Fibroblasts were used to study cell adhesion, spreading, and proliferation.

In 2016, to mimic ECM structure, a PLGA copolymer immobilized with GRGDY peptide was electrospun by Kim et al. to form nanofibers. PLGA-b-PEG-NH_2_ was blended with PLGA as an electrospinning ink to introduce free amino groups, which can be used to functionalize RGD peptides. The polymer concentration and blend ratio influence the characteristics of the nanofiber. Compared with nanofiber without immobilized RDG peptides, those with RGDs greatly enhanced cell adhesion, spreading, and proliferation (Figure 13) [127].

In 2014, to challenge the topic of nerve function recovery, Yun et al. prepared electrospun nanofiber scaffolds by electrospinning a mixed solution of RGD-functionalized poly(serinol-hexamethylene urea) (PSHU-RGD) and poly-ε-caprolactone (PCL). Compared with laminin-coated surfaces, no cytotoxicity of PSHU-RGD/PCL nanofiber scaffolds was found in the MTT test. PSHU-RGD/PCL not only promoted PC12 cell adhesion and differentiation but also enhanced neurite outgrowth [128]. In 2018, Madhavan et al. demonstrated that RGD functionalized PSHU/PCL biomaterial could be also used in vascular grafts [129].

In 2014, to overcome the shortcoming of difficult infiltration of cells into electrospun scaffolds, Jeong et al. proposed that electrospun mats with high porosity, thickness, and small-fiber diameter should be considered. To achieve this purpose, high humidity conditions and ultra-sonication were introduced in the preparation process of RGD-modified alginate mats [130]. In 2016, Antonova et al. reported that electrospinning of a blend of poly(3-hydroxybutyrate-co-3-hydroxy valerate) (PHBV) and poly(caprolactone) (PCL) gave small-diameter biomaterial grafts for vascular tissue engineering. In the rat’s study of grafts implantation, it was found that RGD or VEGF (vascular endothelial growth factor) modified PHBV/PCL grafts promoted endothelialization, collagen production, and primary patency rate [131].

#### 7.4.6. 3D Printing

In 2014, Wang et al. reported that filamentous phage can be genetically fused with RGD peptides to the terminal of the major coat protein pVIII, forming RGD-phage nanofibers. A porous bioceramic scaffold was fabricated using 3D printing technology, then it was immersed into a mixture of negatively charged RGD-phage nanofibers and positively charged chitosan, which allowed to stabilize the phage nanofibers. The scaffold seeded with MSCs and implanted into the defect site of rats, and the formation of new bones containing new blood vessels were observed [132].

**Figure 14:**
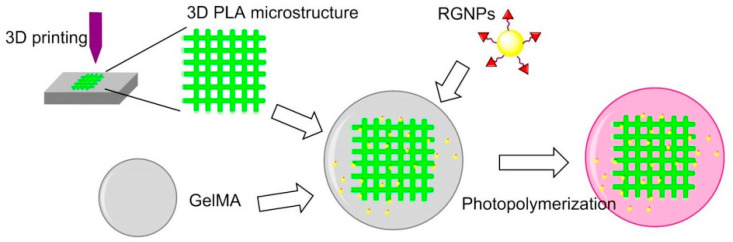
3D printing for surface functionalization. 3D PLA microstructure was prepared via 3D printing technology; GelMA was obtained by methacrylation of gelatin; peptide-conjugated Gold NPs (RGNPs) were prepared by reduction of chloroauric acid before conjugating RGD peptides.

Hydrogel is not suitable for musculoskeletal tissue engineering because of their stiffness. In 2017, Heo et al. found that when cRGD-conjugated gold NPs (RGNPs) were incorporated into the microstructure of 3D printed hydrogel-embedded polylactic acid (PLA), the stiffness can be enhanced to be as strong as native bone tissue. The stiffness could be also modulated to simulate the stiffness of human mandibular condyle. Human adipose-derived stem cells (ADSCs) were encapsulated in reinforced composite hydrogels; it was found that the RGD peptide increased cell adhesion, spreading and proliferation, and osteogenic differentiation (Figure 14) [133].

Unlike modifications after fabrication, in 2019, Chow et al. [134] introduced a 3D printing method that used the biodegradable polymers poly(caprolactone) (PCL) pre-functionalized with RGDs or RGEs (control) to modify the surface. Fibroblasts preferred to attach and spread on RGDS (biotin)-PCL fibers rather than RGES (azide)-PCL fibers, and increasing the concentration of RGDS (biotin)-PCL can promote cell adhesion.

### 7.5. Detection of Tumor Cells

**Figure 15:**
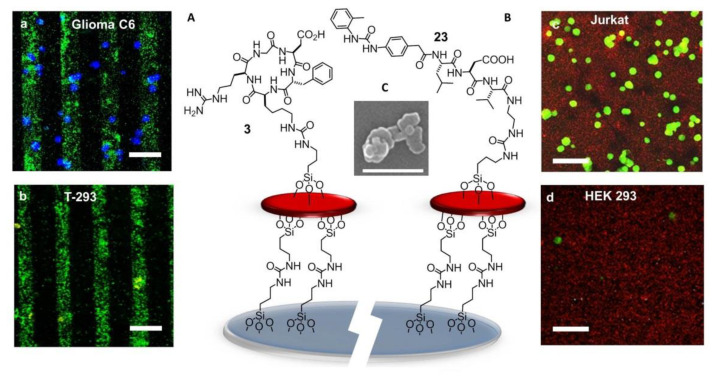
(**A**) Sketch of c[RGDfK]-zeolite SAM bound onto a glass substrate. Confocal images of the patterned c[RGDfK]-SAM after 30 min incubation with (**a**) glioma C6 and (**b**) primary endothelial cells T-293 (scale bar = 100 μm). (**B**) Sketch of urea-LDV-zeolite SAM. Confocal microscopy images of urea-LDV-SAM visualized after 15 min incubation with (**c**) Jurkat cells and (**d**) HEK-293 cells (scale bar = 50 μm). (**C**) SEM image of zeolite L crystals (white bar = 500 nm).

Much effort has also been directed to the preparation of RGD-functionalized bioactive surfaces to favor integrin-mediated cell adhesion and growth. For such uses, long contact times between the substrates and the cells are envisaged. Less attention has been paid to the development of RGD-functionalized bioactive surfaces as diagnostic devices to detect cancer cells, for which a fast and yet selective and strong adhesion is preferable. These devices can be exploited for the entrapment and study of circulating tumor cells (CTCs). These are cells that detach from solid primary tumors during metastasis, entering the blood circulation. The importance of CTC counting in cancer diagnostics has grown over the past decades, as their concentration in the blood represents an indicator of tumor invasiveness, allowing monitoring of the therapeutic outcomes of cancer. In 2015, Greco et al. designed an integrin-targeting nanostructured device constituted of patterned SAMs of disk-shaped zeolite L nanocrystals coated with the cyclic integrin ligand c[RGDfK] (**3**) (Figure 15A) [135]. 

Zeolite SAMs were chosen for the large surface area and the possibility of the high density of superficial functionalization with bioactive molecules, providing a large number of contact points, exploitable for more effective binding to biological systems. Large and ultra-flat disk-shaped zeolite L crystals (about 1000 × 250 nm) were loaded with the fluorescent dye DXP, and subsequently, their surface was functionalized with an isocyanate linker. Then, the NPs were covalently bound onto the silica plated functionalized with amino groups on the surface, by the formation of urea linkages. Patterning into stripes was done to better highlight the specific attachment of the cells only on the peptide-functionalized regions of the zeolites. Once prepared, the printed substrate was coupled with the integrin ligand c[RGDfK]. Adhesion experiments were performed with the integrin-expressing cancer cell lines HeLa and Glioma C6. Confocal microscopy (Figure 15a,b) showed that the population of adherent HeLa and Glioma C6 cells was much higher than that of T-293 cells (negative control).

### 7.6. Ligands Other than RGD 

The next advances in integrin-binding surfaces are likely to emerge from expanding to other ligands than RGD. For instance, a device for the identification and quantification of the leucocytes expressing active α4β1 integrins could be utilized for monitoring ongoing inflammatory activity. For the detection and counting of leucocytes, attention was turned to peptide-coated SAMs of Zeolite L crystals capable to reproduce the cell adhesive multivalency integrin-ligand interaction at the endothelial surfaces in the proximity of the sites of inflammation [136]. The SAMs were coated with α4β1 integrin-targeting Gln–Ile–Asp–Ser (QIDS) sequence, the minimal epitope of the natural ligand VCAM-1, or a peptide ligand derived from the α4β1 integrin ligand MPUPA–Leu–Asp–Val (**23**) (Figure 15B), a sequence shared with the potent antagonist BIO1211 (**7**). This peptidomimetic included the minimal epitope of the natural ligand fibronectin (FN), namely the tripeptide LDV, plus the MPUPA moiety at the N-terminus which strongly increased the α4β1 integrin affinity (IC_50_ of urea-LFV = 30 nM). Cell adhesion experiments were performed in Jurkat cells, an immortalized cell line of human T lymphocytes often utilized as prototypic α4β1 integrin-expressing cells. Confocal microscopy revealed that MPUPA-LDV-SAM showed the highest adhesion of Jurkat cells compared to the negative control HEK-293 cells (Figure 15c,d), 1.4 × 10^4^ versus 1.7 × 10^3^ cells/cm^2^.

Peptidomimetic molecules selective for integrin ανβ3 or α5β1 were utilized for coating bactericidal titanium surfaces reproducing the bactericidal needle-like nanotopography of certain insects’ wings. The functionalization increased MSCs adhesion to the surfaces, and the αvβ3-selective peptidomimetic-coated materials promoted osteogenesis, while the antibacterial activity of the substrates was maintained when tested on pathogenic *Pseudomonas aeruginosa* [137].

β-lactams were also reported to be integrin ligands, especially for integrin αvβ5, α5β1, or α4β1 [50,51,52]. To improve human mesenchymal stem cell adhesion and promote the application in tissue engineering and regeneration medicine, β-lactam-based agonist ligands (see also section “Addressing Integrins with Agonist Ligands”) were incorporated into poly(L-lactic acid) (PLLA) to form functionalized scaffolds by electrospin technology [138].

### 7.7. Multifunctional Integrin-Targeting Biocompatible Surfaces

The ECM is a multifunctional material with multiple components. Thus, biomaterials need to possess multiple features to recapitulate the essential functionality of these ECM components to satisfy the needs of the cells when they are developed for biomedical applications such as tissue engineering and regenerative medicine. To fulfill this requirement, it is important to not only select a biocompatible material as the fundamental structural component of the ECM mimic but also functionalize this material with a biologically active molecule serving biochemical and biophysical cues. In 2011, Shen et al. reported an engineered biomimetic substrate functionalized with both an FN-derived peptide ligand for α5β1 and a CCN1 (or CYR61, Cysteine-rich angiogenic inducer 61)-derived peptide ligand for α6β1 integrins. The surfaces were prepared by immobilizing cysteine- polypeptide ligands on gold-coated slides and supported efficient early mesodermal differentiation of human embryonic stem cells (hESCs) when cultured in a differentiation medium containing BMP4, while mesodermal differentiation was not induced on substrates functionalized with either ligand alone [139].

cRGD and the adhesive peptide sequence PHSRN found in human FN were assembled in a chemically defined and controlled fashion on a peptide-based divalent platform. A Lys-betaAla-Cys sequence was utilized as a branching unit at Lys and as an anchoring group at Cys, to provide a chemoselective, strong and stable binding of the adhesive sequences onto Ti samples. The surfaces coated with the platform of cRGD/PHSRN efficiently supported and promoted good levels of attachment, spreading, proliferation, and differentiation of osteoblast-like cells [140].

An RGD peptide and an anti-VEGF aptamer were incorporate through free radical polymerization into a 3D porous PEG hydrogel to develop a dual-functional biomaterial [141]. The data showed that the dual-functional porous hydrogel enhanced the growth and survival of endothelial cells. The integrin ligand promoted the attachment and growth of endothelial cells in the hydrogel, and the antivascular endothelial growth factor aptamer was able to sequester and release VEGF of high bioactivity.

In 2018, Qiao et al. realized a low-fouling polymeric surface-functionalized with nano-clusters of ligands that bind two receptor types which contribute to FA signaling and mechanotransduction, i.e., integrin and syndecan-4 receptors. The clustered surfaces were generated by film casting blends of highly functionalized polymer chains of methyl methacrylate with PEG pendant chains, with non-functionalized polymer chains. The blending strategy created nano-islands of high peptide density. The presence of both ligand types synergistically increased >2-fold the adhesion HUVEC cells and increases the rate of surface endothelialization compared to surfaces functionalized with only one ligand type. Additionally, the mixed population of ligands was shown to regulate endothelial cell migration and induced the appropriate morphological changes (elongation and alignment in the direction of flow), when exposed to laminar shear flow [142]. 

## 8. Nanostructured 2D or 3D Smart Interfaces for Dynamic Cell Adhesion

Recent efforts have been directed towards nanostructured 2D or 3D materials which can be used as smart interfaces to further understand and control the complex interplay of events and interactions occurring within living cells [97,143]. Smart interfaces were triggered according to cellular microenvironment or stimulation outside to study fundamental cell phenomena or achieve precise and controlled drug delivery.

### 8.1. Thermoresponsive Polymers

**Figure 16:**
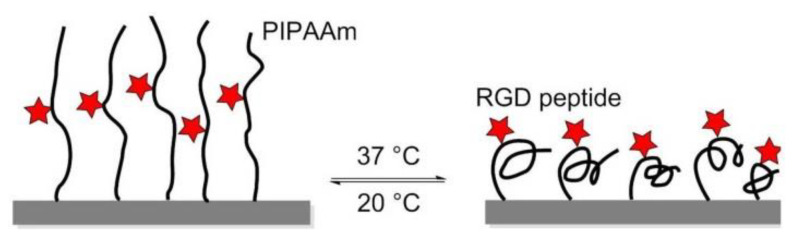
The thermoresponsive PIPAAm polymer undergoes expansion at 20 °C, and the RGD peptide remains hidden into the polymer. At 37 °C, the polymer shrinks and exposes the RGD peptide, promoting cell adhesion.

In 2008, Ebara et al. proposed a thermoresponsive dish to culture cells, to allow the detachment of the cells by simply reducing the temperature, without digestive enzymes or chelating agents. The temperature-responsive polymer poly (N-isopropyl acrylamide) (PIPAAm) and the cell adhesion peptide Arg–Gly–Asp–Ser (RGDS) were bounded to the surfaces. When the temperature was 37 °C, PIPAAm dehydrated and shrunk, causing the RGD peptide to be exposed, thus consenting cell adhesion. When cells were cultured at 20 °C, the grafted PIPAAm layer was hydrated and expanded, so the RGD peptide was shielded by PIPAAm, inducing cells detachment from the surface (Figure 16) [144]. Subsequently, in 2017, Kobayashi et al. reported a system that was optimized by co-immobilization of the Pro–His–Ser–Arg–Asn (PHSRN) peptide found in the 9th type-III repeat of FN, or by the introduction of spacers (PEG, glycine hexamer, streptavidin-biotin) [145]. 

In 2010, Simnick et al. utilized stimulus-responsive elastin-like diblock copolymers (ELPBCs), i.e., genetically encoded polypeptides composed of a Val–Pro–Gly–Xaa–Gly repeat (Xaa is any amino acid besides Pro), that exhibited inverse phase transition behavior at a specific transition temperature. The ELPBCs were exploited as a scaffold to bind and present the GRGDS peptide, an integrin ligand characterized by low affinity (IC_50_ = 1 mM) for the ανβ3 integrin. Despite the low affinity of this ligand, it has been observed that the avidity of K562 human leukemia cells transformed by ανβ3 receptor (K562/ανβ3) is higher as compared with a wild-type K562 control, through multivalent presentation induced by the thermally triggered self-assembly of the ELPBCs [146].

### 8.2. Enzyme-Triggered Polymers

**Figure 17:**
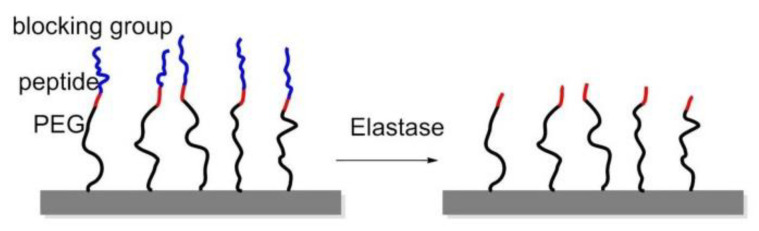
Enzyme-triggered polymers were immobilized on glass. The RGD peptide was blocked by Fmoc, amino acid, or polyethyleneglycol (PEG). Elastase can cleave the blocking group at a specific position thus exposing the RGD peptide, thereby improving cell adhesion.

To manage adhesion/cytoskeletal balance and to initiate differentiation of MSCs to prolong cell culture, in 2016, Robert et al. prepared surfaces immobilized by RGD-containing polymer monolayer, which can be enzyme-activated. This surface consisted of RGD peptide or RGE peptide (control), an elastase cleavable dialanine (AA) linker, and steric blocking fluorenylmethyloxycarbonyl (FMOC) group, or adhesion-reducing PEG. Elastase removed the FMOC/PEG blocking group exposing RGD and generating cell adhesion and differentiation (Figure 17) [147].

### 8.3. Redox-Switchable Polymers

**Figure 18:**
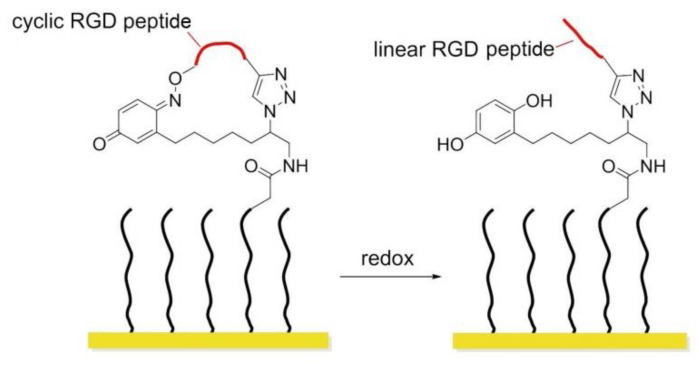
Redox-switchable polymers. In the cyclic form, the RGD sequence is blocked and inactive; after redox, the RGD peptide is exposed.

In 2011, Yousaf and co-workers developed a dynamic hide-and-reveal ligand adhesion strategy for controlling substrate adhesiveness for biospecific cell attachment. First, a building block containing both alkyne and oxyamine functional groups was reacted via Huisgen 1,3-cycloaddition with hydroquinone (HQ)-azide linker, which was already immobilized onto the surface. Mild oxidation converted the HQ to benzoquinone (BQ), and the latter gave intramolecular cyclization via oxime chemistry with the oxyamine group on the ligand. Upon application of a reducing potential to the substrate, the oxime bond was cleaved, regenerating the HQ and exposing the linearized RGD ligand. Fibroblast cells were observed with timelapse microscopy; smaller cells were found to migrate faster upon switching from cyclic to linear RGD. Conversely, a higher degree of actin fiber organization and stronger vinculin localization were observed on cyclic RGD (Figure 18) [148]. 

Later that year, the same author introduced a photo/redox strategy to selectively immobilize ligands in defined areas to control cell adhesion, tissue morphing, and cell migration [149]. HQs on a SAM were uncaged by UV illumination and then electrochemically converted to BQs, which in turn were reactive toward oxyamine-modified RGD ligands. Adhered fibroblasts were first confined to hydrophobic patterns; after installing RGD ligands, cells migrated from the hydrophobic patterns toward the RGD presenting patterns. When a gradient of RGD ligands was formed, cells were observed to move faster toward lower ligand densities instead of higher ones.

### 8.4. Potential Responsive Polymers

In 2012, Gooding and coworkers prepared two-component SAMs comprising a protein-resistant ethylene glycol chain containing a charged moiety at its distal end, and a terminated RGD component on which cellular adhesion receptors, integrins can bind. The electro-switchable surfaces were able to control cell adhesion under different electrical potential. In one case, the surface was constituted by an RGD and hexa(ethylene glycol)-sulfonate species (EG6) mixed SAM that promoted cell adhesion under a positive potential (+0.3 V), while in the other case the surface comprises an RGD and EG5-ammonium mixed SAM that prevents cell adhesion at the same positive potential [150] while promoting cell adhesion at negative potentials (−0.3 V).

**Figure 19:**
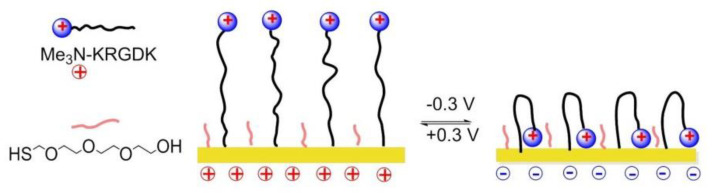
Potential responsive polymers on the Au surface. When the surface is positively charged, positively charged RGD-containing molecule (Me_3_N^+^)-KRGDK is extended, and the RGD peptide is exposed and active. When the surfaced is negatively charged, (Me_3_N^+^)-KRGDK is hidden.

A switchable SAMs based on electrochemical potentials was reported by Zhang and his coworkers in 2015 [151]. Reversible modulation of cell adhesion/migration based on switch of potential because of transformation between linear RGD and cyclic RGD. Two-component SAMs were formed on a gold surface. One of them consisted of cysteine, an ethylene glycol, and an RGD peptide (Me_3_N^+^)-KRGDK with a positively charged quaternary ammonium group. The other one was a thiol group headed tetra ethylene glycol (TEG) for preventing nonspecific cell adhesions. When the surface was positively charged (+0.3), the distal end of RGD-NMe_3_ with positive charge were repelled by electrostatic repulsion, to form a linear RGD domain capable to interact with cells. On the contrary, under a negative potential (−0.3 V), the (Me_3_N+)-KRGDK component was hidden into the TEG due to the electrostatic attraction (Figure 19).

### 8.5. Photo Responsive Polymers

In 2013, Surfaces modified with photo-activable adhesion peptides were designed by Salierno et al. as platforms for advanced cell migration studies. The surfaces were functionalized with the 3-(4,5-dimethoxy-2-nitrophenyl) butan-2-ol (DMNPB) modified peptide c[RGD(DMNPB)fK]. Cell attachment and migration were independently triggered by subsequent light exposure steps. Indeed, RGD adhesive patterns were generated after irradiation to cleave the Asp-DMNPB ester in specific areas. Subsequent cell seeding gave monolayer formation onto the exposed zones, and a second irradiation step activated further migration [152].

**Figure 20:**
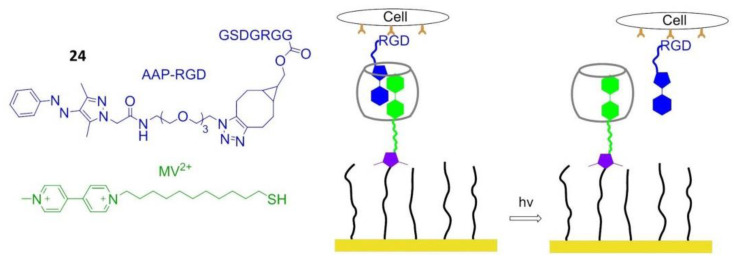
Photo-responsive polymers. AAP-RGD is isomerized from E- to Z-isomer under UV irradiation at 365 nm, resulting in dissociation of AAP-RGD from MV^2+^/AAP-RGD/CB[8] complex. When AAP-RGD is irradiated at 520 nm, the E-isomer is reobtained, and the MV^2+^/AAP-RGD/CB[8] complex can reform.

In 2018, Wiemann et al. reported that in the presence of methyl violet (MV^2+^), the photo-convertible arylazopyrazole (AAP) derivative could be encapsulated in cucurbit[8]uril (CB[8]) to form a hetero ternary photoswitchable complex. Under the irradiation of light with a wavelength of 365 or 520 nm, AAP was transformed from E- to Z-isomer or vice versa, and the Z isomer caused the dissociation of the complex. When the Arg–Gly–Asp–AAP (AAP-RGD) peptide (**24**) was attached to a CB[8]/MV^2+^ complex bounded biologically active surface, cell adhesion, and release could be controlled by UV radiation (Figure 20) [153].

### 8.6. Electrochemically Controlled Polymers

**Figure 21:**
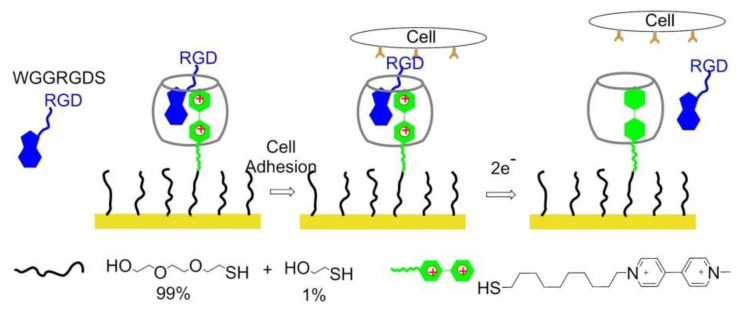
Electrochemically controlled polymers on the surface. The WGG-viologen-CB[8] complex is immobilized on the gold surface when viologen gets 2 electrons, and WGGRGDS is released from the complex.

Cucurbit[8]uril (CB[8]), a macrocyclic host molecule capable of binding two aromatic guest molecules simultaneously, was utilized for the preparation of electrochemically controlled noncovalent functionalized cell-adhesive surfaces. One guest was Trp–Gly–Gly (WGG)-RGDS, the second one was electroactive viologen modified with an alkyl thiol group to provide a bond with gold slides. The ternary WGG-viologen-CB[8] complex containing the RGDS ligand to recognize integrin receptors and mediate cell adhesion. Subsequently, electrochemical reduction of viologen leads to the release of the peptide and subsequent detachment of cells from the slides (Figure 21) [154].

### 8.7. Dynamically Competitive Polymers

**Figure 22:**
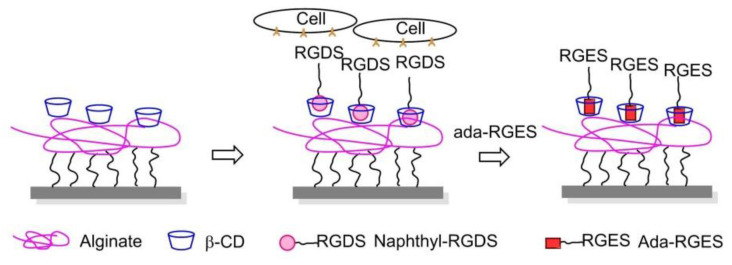
Dynamically competitive polymers. Guest molecular naphthyl-RGDS is captured by β-CD, which is grafted on alginate. Cell adhesion and spreading can be controlled dynamically because the addition of Ada-RGES can reverse cell adhesion due to it competed with naphthyl-RGDS to form a complex with β-CD.

In 2013, Stupp and coworkers engrafted β-cyclodextrin (β-CD) to alginate, a well-known non-spreading surface for 3T3 fibroblasts, and the CD-alginate was stamped to glass. The addition of soluble 1-naphthoic acid-pentaglycine (G5) linker-RGDS guest molecules to the culture media induced FA formation and cell spreading thanks to the naphthyl host-CD guest interactions. The spreading of cells on the substrate was reversed by the addition of adamantane-RGEs, which are competitive guest molecules, supporting that the expressed cue on this artificial ECM can be controlled dynamically (Figure 22) [155].

### 8.8. Dynamically Controlled Smart Interfaces for 3D Cell Culture

The dynamic cell-instructive biointerfaces represent a newly proposed concept as smart interfaces, aimed at mimicking native 3D microenvironment that surround encapsulated cells to implementation of sufficient complexity and dynamism to instruct cells toward a certain behavior. Multiple 3D biomaterial strategies have been developed based on synthetic polymers and natural ECM (e.g., proteins and polysaccharides). Advanced 3D dynamic cell-culture platforms for regenerative medicine applications took advantage of nondegradable PEG hydrogels.

**Figure 23:**
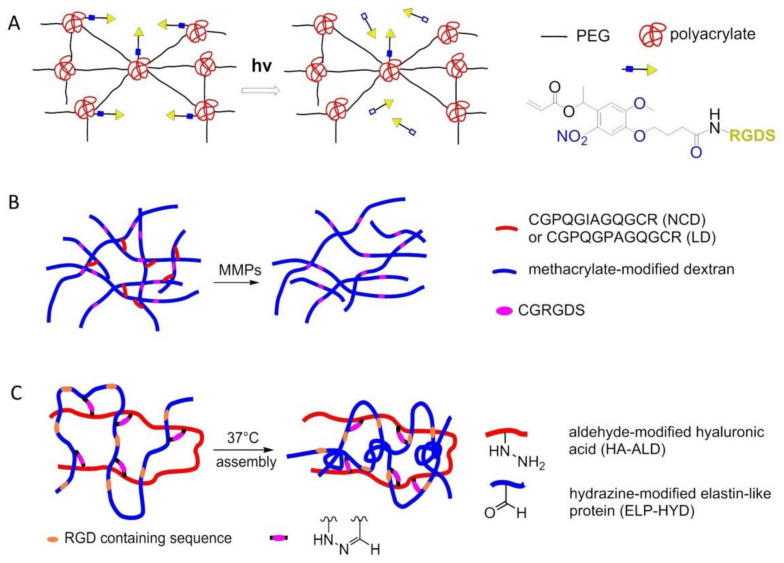
Smart interfaces for 3D cell culture. (**A**) Poly(acrylate)-PEG 3D gel containing RGD peptide and photocleavable domain. (**B**) ELP-HA hydrogel containing RGD sequence. (**C**) DexMA hydrogels functionalized RGD peptide.

In 2009, Kloxin et al. exploited photodegradable linkages to locally modify the chemical environment within a hydrogel. A biofunctional acrylic monomer containing the adhesion peptide RGDS was attached to a photodegradable acrylate. The monomer was polymerized into a nondegradable PEG gel. The chemical composition of the resulting poly(acrylate)-PEG 3D material was controlled with light exposure by photolytic release of the tethered biomolecule RGDS. hMSCs were encapsulated until the presentation of RGDS was temporally altered by photocleavage of RGDS from the gel on day 10 in culture. RGDS presentation maintained hMSC viability within PEG-based gels while RGDS photolytic removal directed hMSC chondrogenic differentiation, indicating that the cells responded to the chemical change in their environment (Figure 23A) [156].

In 2017, Trappmann et al. introduced 3D hydrogels of methacrylated dextran (DexMA) functionalized with RGD and crosslinked with collagen-derived peptides that can be cleaved enzymatically by cell-secreted matrix metalloproteinases (MMPs), have been used in the context of an in vitro study of angiogenic sprouting (Figure 23B) [157]. Another strategy to introduce dynamicity to hydrogel design is to make use of covalent bonds that can be formed, broken, and reformed under equilibrium conditions. In the same year, Heilshorn and co-workers introduced a thermoresponsive engineered elastin-like protein (ELP), incorporated with a cell-adhesive RGD sequence, to develop a novel dual-crosslinked adaptable hydrogel with improved material properties for injectable cell delivery. A mixture of hydrazine-modified ELP (ELP-HYD) and aldehyde-modified hyaluronic acid (HA-ALD) macromers was used at room temperature to induce the first gelation. Upon heating to physiological temperatures, mechanical properties of the hydrogel improved, enabling easy injectability of encapsulated hMSCs (Figure 23C) [158].

## 9. Conclusions

This paper aimed at presenting the more recent uses of integrin-targeting peptides for biofunctionalization of diverse bulk materials or nanomaterials. Micelles, dendrimers, paramagnetic liposomes, chitosan NPs, silicon NPs, Au NPs, iron oxide NPs, quantum dots (QDs), etc. have found applications not only to deliver anti-tumor drugs, but also as diagnostic probes, or to deliver proteins and siRNA, for the detection and treatment of tumors and other severe diseases. Besides, using appropriate linkers, integrin-targeting peptides can be anchored onto a variety of organic or inorganic nanomaterials, surfaces, or polymers, etc., by forming covalent or non-covalent bonds. Interestingly, cleavable covalent bonds or non-covalent interactions have been utilized to deliver cargos more precisely under the stimulation of certain conditions. More recently, the peptides have been combined with smart materials to obtain thermally-responsive, redox-controlled, light-sensitive, potential-controlled, smart nanomaterials for controlling cell adhesion. Host-guest interaction molecules can be exploited to realize light or chemically regulated, reversible cell adhesion. In summary, these peptide-material conjugates might consent applications ranging from fundamental cellular studies to oncology diagnostics, orthopedic biomaterials, regenerative medicine, tissue engineering, and other fields.

So far, the large majority of the peptide-functionalized materials are utilized as integrin-targeting molecules cyclic or linear peptides containing the RGD sequence. In perspective, other integrin-binding sequences such as the LDV, QIDS, etc., as well as recently discovered agonist ligands, are expected to gain increasing attention in the next future.
